# Current Status and Future Directions of Neuromonitoring With Emerging Technologies in Neonatal Care

**DOI:** 10.3389/fped.2021.755144

**Published:** 2022-03-23

**Authors:** Gabriel Fernando Todeschi Variane, João Paulo Vasques Camargo, Daniela Pereira Rodrigues, Maurício Magalhães, Marcelo Jenné Mimica

**Affiliations:** ^1^Division of Neonatology, Department of Pediatrics, Irmandade de Misericordia da Santa Casa de São Paulo, São Paulo, Brazil; ^2^Clinical Research Department, Protecting Brains and Saving Futures Organization, São Paulo, Brazil; ^3^Division of Neonatology, Grupo Santa Joana, São Paulo, Brazil; ^4^Data Science Department, OPD Team, São Paulo, Brazil; ^5^Pediatric Nursing Department, Escola Paulista de Enfermagem, Universidade Federal de São Paulo, São Paulo, Brazil; ^6^Department of Pediatrics, Faculdade de Ciências Médicas da Santa Casa de São Paulo, São Paulo, Brazil; ^7^Department of Pathology, Faculdade de Ciências Médicas da Santa Casa de São Paulo, São Paulo, Brazil; ^8^Department of Pediatrics, Irmandade da Santa Casa de Misericórdia de São Paulo, São Paulo, Brazil

**Keywords:** amplitude-integrated electroencephalography, near-infrared spectroscopy, multimodal monitoring, telemedicine, artificial intelligence, machine learning

## Abstract

Neonatology has experienced a significant reduction in mortality rates of the preterm population and critically ill infants over the last few decades. Now, the emphasis is directed toward improving long-term neurodevelopmental outcomes and quality of life. Brain-focused care has emerged as a necessity. The creation of neonatal neurocritical care units, or Neuro-NICUs, provides strategies to reduce brain injury using standardized clinical protocols, methodologies, and provider education and training. Bedside neuromonitoring has dramatically improved our ability to provide assessment of newborns at high risk. Non-invasive tools, such as continuous electroencephalography (cEEG), amplitude-integrated electroencephalography (aEEG), and near-infrared spectroscopy (NIRS), allow screening for seizures and continuous evaluation of brain function and cerebral oxygenation at the bedside. Extended and combined uses of these techniques, also described as multimodal monitoring, may allow practitioners to better understand the physiology of critically ill neonates. Furthermore, the rapid growth of technology in the Neuro-NICU, along with the increasing use of telemedicine and artificial intelligence with improved data mining techniques and machine learning (ML), has the potential to vastly improve decision-making processes and positively impact outcomes. This article will cover the current applications of neuromonitoring in the Neuro-NICU, recent advances, potential pitfalls, and future perspectives in this field.

## Introduction

Despite recent breakthroughs in perinatal care, impaired outcomes in newborns at high risk for brain injury remain common, posing a challenge in neonatal care and public health (1, 2).

Neonatal encephalopathy (NE) and perinatal stroke in term infants, as well as the repercussions of germinal matrix-intraventricular hemorrhage (IVH) and white matter injury (WMI) in extremely low birth weight preterm infants, are the most frequent illnesses. Encephalopathy and seizures may also occur due to metabolic and uncommon genetic disorders. All these infants are at high risk of developing neurological impairment, including cerebral palsy, cognitive delay, epilepsy, and other neurological disabilities (3).

No single intervention can reduce newborn brain injury because of the complex interactions between pathological processes, such as inflammation, oxidative stress, developmental trajectory, genetic susceptibility, and environmental influence. However, new approaches focusing on early diagnosis of brain injury together with neuroprotective strategies may improve outcomes. This philosophy has motivated the creation of the neonatal neurocritical intensive care unit (Neuro-NICU) model (4). One of the critical approaches of a Neuro-NICU is continuous brain monitoring, which has been increasingly used to assess brain health in neonates. Continuous EEG (cEEG), amplitude-integrated electroencephalography (aEEG), and near-infrared spectroscopy (NIRS) are non-invasive techniques used at the bedside to evaluate brain function, screen for seizures, and measure regional tissue oxygenation.

The rapid advancement of technology in the NICU may combine the increasing use of telemedicine and artificial intelligence to potentially improve decision-making processes and impact patient outcomes (5, 6). Using these techniques in an integrated manner, also known as multimodal monitoring, may allow practitioners to better understand the physiological processes occurring in critically ill neonates (7). This article will discuss the current applications of neuromonitoring and recent advancements, potential pitfalls, and future perspectives in this field.

## Neonatal Brain Injury

Gestational age continues to be the most critical factor in determining the long-term effects of brain injury, which is complex and multifactorial (8). WMI is the most common type of brain injury in preterm newborns, and it can be found in up to 50% of infants born with very low birth weight (9, 10). However, the spectrum of periventricular WMI has shifted from the severe and commonly cystic lesions toward a milder pattern of WMI as a result of improvements in neonatal care (11). In extremely preterm population, the incidence of all grades of IVH ranges from 31 to 36%, while severe IVH has an incidence between 10 and 17% (12–14). Although brain injury is not always the primary event in the life of a preterm infant, the consequences of preterm birth and associated comorbidities have a significant impact on brain development.

In term infants, perinatal asphyxia and hypoxic-ischemic encephalopathy (HIE) represent the most common cause of brain injury and disability, with an incidence ranging from 1 to 2 per 1,000 live births in high-income countries and significantly higher in low-resource settings (15, 16). Despite being commonly under-diagnosed in the NICU, perinatal stroke has an incidence of approximately 2.5 per 1,000 live births. It is the second most common cause of neonatal seizures and the most common cause of childhood hemiplegia (17).

## Strategies for Neuroprotection and the Neuro-NICU Concept

Following decades of concentrated efforts to improve neonatal intensive care and newborn survival, the focus has shifted to examining the long-term outcomes in this population.

As the standard treatment for infants with HIE, cooling is a significant advance in the field and has effectively reduced the combined risk of death or disability (18, 19). However, there are also several different neurological diseases present throughout the neonatal period, in which early recognition and diagnosis may have a significant impact on disease progression.

The interdisciplinary approach to newborn brain injury care is not new, with numerous units collaborating closely with pediatric neurologists, neurosurgeons, and other professionals. However, the introduction of therapeutic hypothermia represented a paradigm shift that required a concerted approach to identifying and managing patients with NE. The University of California, San Francisco (UCSF) was the first unit in the United States to establish a neonatal neurointensive care nursery (4). This strategy has acquired significant popularity globally during the last decade, with several teams describing their experience establishing similar models of care (20–22).

The objective is to combine advances in neuromonitoring and imaging with novel treatments to enhance neurodevelopmental outcomes. Numerous patient subgroups were identified as possibly benefiting from this therapy approach: term infants with HIE and seizures, extremely low birth-weight preterm infants, and newborns with congenital or uncommon neurological disorders. Additionally, four strategies were developed during the creation of Neuro-NICU programs: (i) infant co-management; (ii) standardized protocols and care bundles; (iii) wider neuromonitoring and neuroimaging utilization; and (iv) establishment of training programs (23–25).

## The Existing Neuromonitoring Tools in the NICU

### aEEG and cEEG

The use of aEEG represents a non-invasive, bedside, and simplified method of continuous brain monitoring, mainly accessed by the neonatologist, which has been increasingly used to assess brain function. Similar to cEEG, it records differences in electrical potentials and changes in electrical activity displayed over time. Then, after being filtered for frequency, the activity is time-compressed, rectified, smoothed, and displayed in a 6 cm/h chart semi-logarithmically.

Compared with the cEEG, the aEEG has the advantage of being more straightforward and quicker to apply. It can be used to evaluate the background activity, sleep-wake cycling, and seizure screening in critically ill neonates as a continuous bedside monitor (26–31).

Persistent pathological background activity and absence of sleep-wake cycling in the first days/weeks of life were associated with higher risk of neurological impairment at 2 years of age in infants with HIE and the preterm population (28, 32–34). On the other hand, the presence of continuous background activity and sleep-wake cycling within the first postnatal week has been associated with a good neurodevelopmental outcome (33).

Klebermass et al. study evaluated 143 preterm infants with gestational age below 30 weeks during the first 2 weeks of life. The aEEG was classified into a graded score according to background activity, appearance of sleep-wake cycling, seizure activity and correlated with neurological outcomes at 3 years of age. Specificity and sensitivity were 95 and 83% respectively in aEEG findings in the second week of life and were superior compared to cranial ultrasound findings (34).

The neonatal period, particularly the first week after birth, is the most susceptible time of human life for seizure development. Seizures are commonly related to acute brain injury, are associated with increased mortality and impairment, and may constitute a neurological emergency, making monitoring and accurate seizure identification critical components of newborn intensive care management (35–37). Higher seizure burden in the neonatal period is commonly associated with abnormal neurological outcomes (38). A clinical trial conducted by Srinivasakumar et al. compared newborns with HIE treated for electrographic seizures with newborns treated only for clinical seizures. The authors found that the treatment of electrographic seizures reduced the seizure burden, severity of MRI findings and improved neurodevelopmental outcomes (39).

Several studies have shown that using two-channel aEEG associated with raw EEG interpretation improves seizure detection accuracy (26, 35–37, 40–44). Seizure activity is characterized by a sudden change on aEEG background activity as an abrupt rise in minimum and maximum amplitudes correlated with an evolving, stereotypical, and rhythmic wave pattern, with repeating forms such as spikes or sharp waves seen in raw EEG, with a total duration of at least ten seconds (45). The aEEG seizures are classified into (a) single seizures (SS): one electrographic seizure per each epoch; (b) repetitive seizures (RS): more than one electrographic seizure per epoch but less than one electrographic seizure over a 10 min period; (c) status epilepticus (SE) in the neonate. SE is defined by: (a) continuous seizure activity for at least 30 min or (b) recurrent seizures for over 50% of the recording time ranging from 1 to 3 h (42).

**Figure 1:**
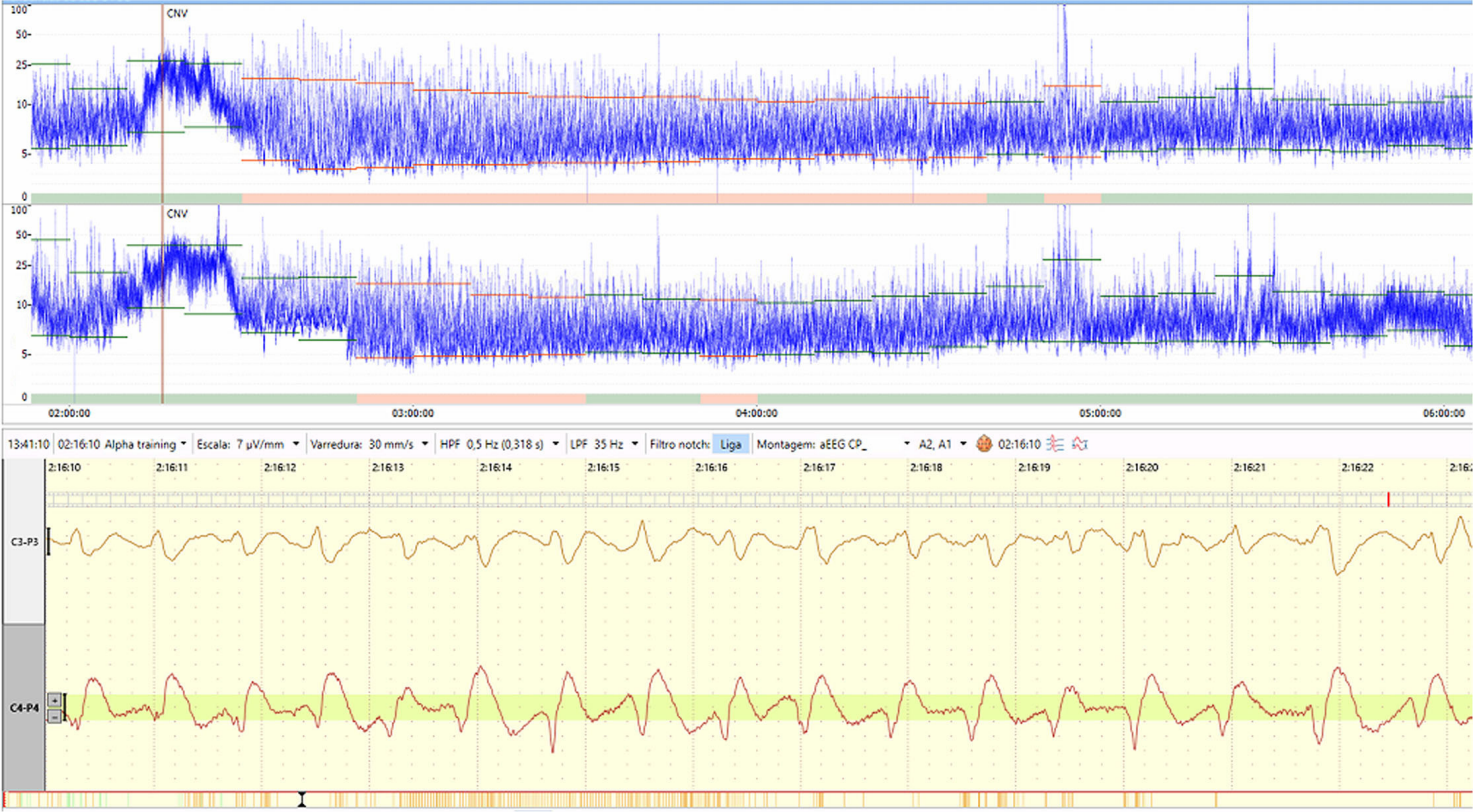
An example of seizures displayed on two-channel aEEG and raw EEG. Figure from the authors' personal file.

Figure 1 displays an example of seizures shown on two-channel aEEG and raw EEG.

Other high-risk populations, such as infants born with congenital heart diseases (CHD), stroke, metabolic disorders, inborn errors of metabolism (IEM), cerebral malformations, and congenital infections, may benefit from this screening tool (46–51). Gunn et al. evaluated a large cohort of 150 newborn infants undergoing cardiac surgery who underwent aEEG monitoring in the perioperative period (prior to and during surgery, and for 72 h postoperatively). Perioperative electrical seizures were found in 30%, of whom 1/4 had any clinical correlation. Failure to recover to a continuous background aEEG by 48 postoperative hours was associated with impairment in all outcome domains and abnormal aEEG at seven postoperative days was highly associated with mortality (46).

The cEEG is a noninvasive procedure performed using electrodes attached to the scalp surface in accordance with the international 10–20 system (47) and is the gold standard tool for seizure diagnosis in the NICU (48). The American Clinical Neurophysiological Society (ACNS) has published recommendations on the use of full-montage electroencephalography (52). Although the idea of the Neuro-NICU has been around for over a decade, the use of extended cEEG is not widespread, with only between 35 and 67% of NICUs reporting its use, indicating an implementation problem (18).

### NIRS

NIRS is a non-invasive technique for monitoring regional tissue oxygenation continuously at the bedside. It can be used as a trend monitor in critically sick newborns to assess the balance of tissue oxygen delivery and consumption, providing cerebral and somatic oximetry readings and enabling early detection of hemodynamic alterations (53). Near-infrared light emitted from a light source on a sensor penetrates the infants' skin and tissue. It is partially absorbed by oxygenated and deoxygenated hemoglobin before being reflected to a detector on the same sensor. After that, a tissue saturation level (rSO_2_) is determined based on arterial to venous blood (25:75) ratio and the balance of oxygen delivery and consumption in the underlying tissue.

Regional cerebral oxygen saturation (rScO_2_) may be easily measured with a sensor placed on the forehead, and readings are verified against jugular venous saturations in newborns (54). The interpretation of rScO_2_ readings requires consideration of other factors that may impact cerebral blood flow and oxygenation, including systemic oxygenation (SpO_2_), cardiac output, anemia, carbon dioxide (CO_2_) tension, glucose levels, and metabolic demand. In an extensive multi-center investigation of the preterm population, normal rScO_2_ levels were shown to range between 55 and 85% (55). Cerebral fractional tissue oxygen extraction (cFTOE) represents the balance between oxygen supply and consumption and is computed using the formula cFTOE = (SpO_2_ – rScO_2_) / (SpO_2_) (56).

Sustaining rScO_2_ levels below 40–50% has been shown to cause cellular, physiological, biochemical, and neuroradiographic brain damage in animal models and infant studies. Hippocampal histology revealed that hypoxic piglets exposed to rScO_2_ 40% for at least 30 mins showed mitochondrial damage and signs of fragmented cellular structure (57). In a study of newborns with hypoplastic left heart syndrome who underwent a Norwood surgical procedure, those with rScO_2_ <45% for over 180 cumulative minutes had a higher risk of developing new or progressive ischemic abnormalities on brain magnetic resonance imaging (58).

In order to implement a NIRS neuromonitoring program, a minimum rScO_2_ threshold and management protocol must be established, so the healthcare team can provide interventions to avoid long-term injury. Causes of abnormal cerebral oxygen supply or demand need to be investigated, including hypocarbia, hypotension, anemia, and low arterial saturation. High tissue oxygen demand in infants with seizures, pain, fever, or infections may also decrease rScO_2_ values.

Several studies highlight the value of two-site NIRS monitoring with brain and somatic measures. Common sites for somatic monitoring include renal or mesenteric/splanchnic regions. Renal regional oxygen saturation (rSrO_2_) values are measured by sensor placement on the posterior flank below the costal margin and above the iliac crest. rSrO_2_ values are sensitive to compromise of systemic blood flow and usually are 10–15% higher than cerebral saturation (59–61). Due to preservation of the cerebral autoregulatory mechanism, hemodynamic compromise typically results in an earlier reduction of somatic oxygenation prior to changes in rScO_2_ (60). Mesenteric regional saturations (mScO_2_) are significantly lower in preterm infants and may fluctuate considerably from 32 to 82 percent (62). Due to its higher variability and more challenging interpretation, mScO_2_ is most commonly applied in research settings.

NIRS monitoring has been shown to be an effective biomarker for early organ dysfunction in many conditions occurring in neonates admitted to the NICU. Additionally, early investigations associated NIRS results with worse short- and long-term outcomes in severely ill infants (62, 63). NIRS has been reported as a valuable technique for monitoring patients following stage 1 palliation for hypoplastic left heart syndrome (HLHS) and demonstrated to improve prediction of neurodevelopmental outcomes at two years of age in newborns with CHD (63, 64). Preoperatively, babies with HLHS who were monitored with NIRS required less mechanical ventilation and inspired nitrogen gas than a historical cohort who were not followed with NIRS (65).

Similarly, in the preterm population, the SafeBoosC consortium recently completed a phase II randomized controlled trial. It examined extremely preterm infants during their first 72 h of life and established the feasibility and efficacy of implementing NIRS continuous monitoring in conjunction with a dedicated treatment guideline (66). Compared to the control group, infants in the NIRS-monitored group exhibited a reduced prevalence of cerebral hypoxia or hyperoxia. Although the study was not powered to detect differences in clinical outcomes, the NIRS-monitored group also tended toward reduced mortality and severe intraventricular hemorrhage.

Also, NIRS may be useful to verify the presence of a hemodynamic significant patent ductus arteriosus (hsPDA). A hsPDA contributes to low cerebral blood flow (CBF) during left to right shunting early in transition when the left ventricle cannot compensate (67). The renal oxygen saturation is decreased and a higher variability is seen. Chock et al. (68) found that renal saturation (rSrO_2_ < 66%) was associated with the presence of an hsPDA and studies were able to detect an increase in cerebral and renal saturation after successful treatment for PDA closure (67). A systematic review conducted by Prescott et al. concluded that NIRS could help clinicians identify hsPDAs and provide information on organ perfusion that may guide treatment decisions (67).

Previously, studies compared rScO_2_ and cFTOE findings before and after drainage, in newborns with post hemorrhagic ventricular dilatation (PHVD). The authors concluded that PHVD is associated with a significant decrease in rScO_2_ and increase in cFTOE due to a decrease in cerebral perfusion. Therefore, NIRS could potentially be a useful biomarker to determine the optimal time point for ventricular decompression (69, 70).

NIRS monitoring has been shown to be useful to evaluate tissue oxygenation in a set of clinical scenarios in the NICU. However, more extensive studies are needed to establish that NIRS monitoring effectively improves outcomes.

## Multimodal Brain Monitoring

According to research in adults and children, multimodal monitoring facilitates the diagnosis and prevention of neurological repercussions in the setting of hemodynamic compromise (71). Simultaneous use of NIRS and aEEG may aid in comprehending the physiology of hemodynamic changes and the risk of cerebral injury.

Several studies described their correlations, especially in HIE. For cooled infants, an abnormal aEEG at 48–72 h and higher tissue oxygenation measured by NIRS at 12–24 h of life is associated with adverse outcomes (62, 72–74). In a cohort of 39 term infants with HIE, Lemmers et al. investigated the predictive value of dual-use of aEEG and NIRS. Combining NIRS and aEEG data enhanced the positive predictive value (PPV) (NIRS 67 % and aEEG 62% vs. combined 91%) and negative predictive value (NPV) (73 and 100% vs. 100%) at 12–18 h of age when compared to either modality alone (62).

Other conditions may also benefit from this combined approach (74). Our group recently published a case review study for this integrated approach in four clinical scenarios, including HIE, hemodynamic instability, patent ductus arteriosus, and seizures (7). Several studies also showed that brain monitoring with aEEG and NIRS is feasible in the delivery room and may provide information on newborns' condition during immediate transition and resuscitation (75, 76). In the Neu-Prem Trial, Katheria et al. evaluated 127 newborns under 32 weeks of gestational age to determine whether NIRS and aEEG can predict infants at risk for IVH and death in the first 72 h of life. The aEEG was not predictive of IVH or death, but NIRS may be used to predict severe IVH and early death (77). Those 127 evaluated newborns were followed out between 22 and 26 months of corrected age in the follow up clinic to assess for neurodevelopmental impairment or death. Authors found that increased duration of hypoxia in infants born under 32 weeks was associated with neurodevelopmental impairment or death (78).

A published recommended neuromonitoring approach for newborns in the NICU is displayed in Table 1. The duration of each brain monitoring approach should be related to periods of higher risk for seizures and infants' clinical instability.

**Table 1:** Neuro-NICU eligibility and recommended neuromonitoring [adapted from Van Meurs et al. (20)].

**Diagnosis**	**Monitoring**
1. HIE/cooling	aEEG, cEEG
2. Seizures	aEEG and cEEG
3. ECMO/pre-ECMO	NIRS and consider aEEG
4. Grade III/IV IVH or PHVD	aEEG
5. Critical/unstable	NIRS and consider aEEG
6. Preterms <28 weeks	aEEG and NIRS
7. CNS anomalies cEEG	cEEG and/or aEEG
8. Metabolic disease	cEEG and/or aEEG
9. Cyanotic CHD	NIRS
10. CNS infection	cEEG and/or aEEG
11. Symptomatic PDA	NIRS
12. ALTE/BRUE	aEEG
13. Hyperbilirubinemia > 20 or hemolytic process	NIRS and consider aEEG

*ALTE, apparent life-threatening event; BRUE, brief resolved unexplained events; CNS, central nervous system; ECMO, extracorporeal membrane oxygenation; PDA, patent ductus arteriosus*.

A multimodal approach can combine these dual brain monitoring techniques with other vital signs and clinical information to study systemic and cerebral hemodynamics and electrographic findings early after birth, allowing a better understanding of the critically ill infant physiology.

A high degree of illness severity, along with limited handling to prevent oscillations in cerebral blood flow, may make multimodal monitoring challenging to apply. Additionally, the small head size, delicate skin, ventilatory support, and high ambient incubator humidity may complicate sensor placement. However, Deshpande et al. presented an early study using a multimodal strategy to examine systemic and cerebral hemodynamics and electrical changes shortly after birth using echocardiogram (ECHO), NIRS, and aEEG to identify infants at risk of IVH. This study established that this is a safe and well-tolerated technique associated with a low risk of adverse events (79).

**Figure 2:**
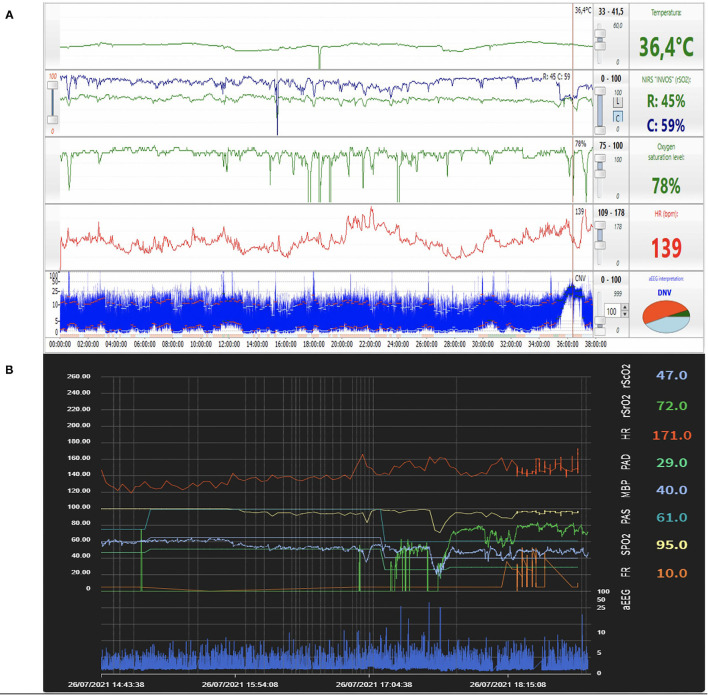
**(A)** Example of a multimodal approach combining aEEG, NIRS, pulse oximetry, heart rate, and temperature findings in real-time. During the seizure episode noted on aEEG (arrow), a decrease in rScO_2_ was observed together with significant fluctuations in pulse oximetry and heart rate. Figure from the authors' personal file. **(B)** Example of a multimodal approach combining aEEG, NIRS, pulse oximetry, heart rate findings in real-time in proprietary software developed using a specific programming language (Python). Correlation between aEEG (continuous low voltage pattern) and low rScO_2_ is observed. Figure from the authors' personal file.

Physiologic information collected by multimodal monitoring may enable the development of neuroprotective strategies. Algorithms for treatment may be based on measures for preventing cerebral hypo- and hyperperfusion. Such an approach may include oxygen delivery modification, ventilatory changes to avoid hypo- or hypercarbia, increasing cardiac output by volume expansion or inotropes, keeping hemoglobin concentrations within a particular range, and PDA closure. Figures 2A,B represent examples of a multimodal approach combining different parameters.

Existing technology for brain monitoring in the NICU, mainly multimodal approach, provides a substantial quantity of data. Data must be stored in a suitable and safe system, with efficient processing and can be integrated with another system or equipment. The improvement of technological capacity generates a number of research possibilities and new perspectives to potentially improve the quality of care.

## Data Integration and Processing

Commercially distributed aEEG/EEG and NIRS monitors are designed primarily for clinical application rather than long-term storage and analysis. Data collection is usually a secondary issue that requires extra planning, infrastructure, and financial resources.

The basics of data capture systems include the following: (a) synchronized gathering of vital signs, (b) precise time/date information, and (c) data loss prevention. Whatever technique is chosen, data is essential and should be collected at the greatest sampling rate feasible (80, 81).

### Direct Download From a Local Device

The easiest method of obtaining data from brain monitoring equipment is by direct download. While this is the simplest method, it does have several disadvantages. Data is only accessible following the conclusion of a monitoring session. Data loss can occur as a consequence of power failure or unintentional deletion from the device. The limited memory capacity of each device may result in data files being overwritten if not retrieved on a regular basis. Second, this technique allows collecting a single monitoring data, and other vital signs are commonly omitted from the file. As a result, the data collected in this method must be externally reconciled with other vital signs data. Finally, the timestamp for this monitor data comes from the monitor's clock, which is frequently not updated for daylight savings time or is reset in a power failure.

### Data Collection Through a Central Hub and Central Server

A central computer is required to coordinate the synchronized acquisition of physiological signals from numerous sources. Typically, a central-hub method involves placing a laptop computer at the patient's bedside directly linked to the patient monitors (including aEEG/EEG, NIRS, and vital signs monitor), the ventilator, or other equipment. This technique is helpful for time syncing because the computer supplies the timing data, and the program writes the data straight to disk, ensuring that previously recorded data is not lost. Additionally, some software packages offer integrated analytic tools and can serve as an accessible open path for inexperienced investigators. The primary downside of the central-hub model is that it typically needs extra hardware (laptops, computer, cabling) or software purchases and may impose limits due to device compatibility limitations.

The most commonly used protocols for data export and integration are Lab Stream Layer (LSL)—for EEG equipment—and Health Level Seven (HL7)—widely used in medical devices (e.g., vital signs monitor, electronic records, ventilators) (82, 83). Many other protocols are used by different devices, some unique according to the model and manufacturer. For this reason, when gathering data from multiple types of equipment, a tool to convert each data input into a structured format is needed. Programing languages like Python and Matlab are commonly used for the development of these tools (84–86).

A variation on this data collection approach is the central-server concept, in which separate monitoring devices are linked to a centrally placed server through a network. The technique retains the benefit of synchronized timing and adds the use of primarily automatic capture, requiring minimum human input to start and stop the recording and eliminating the requirement for a laptop at each patient's bedside. The main drawback is that it is the most complicated infrastructure option, frequently necessitating substantial capital expenditure.

**Figure 3:**
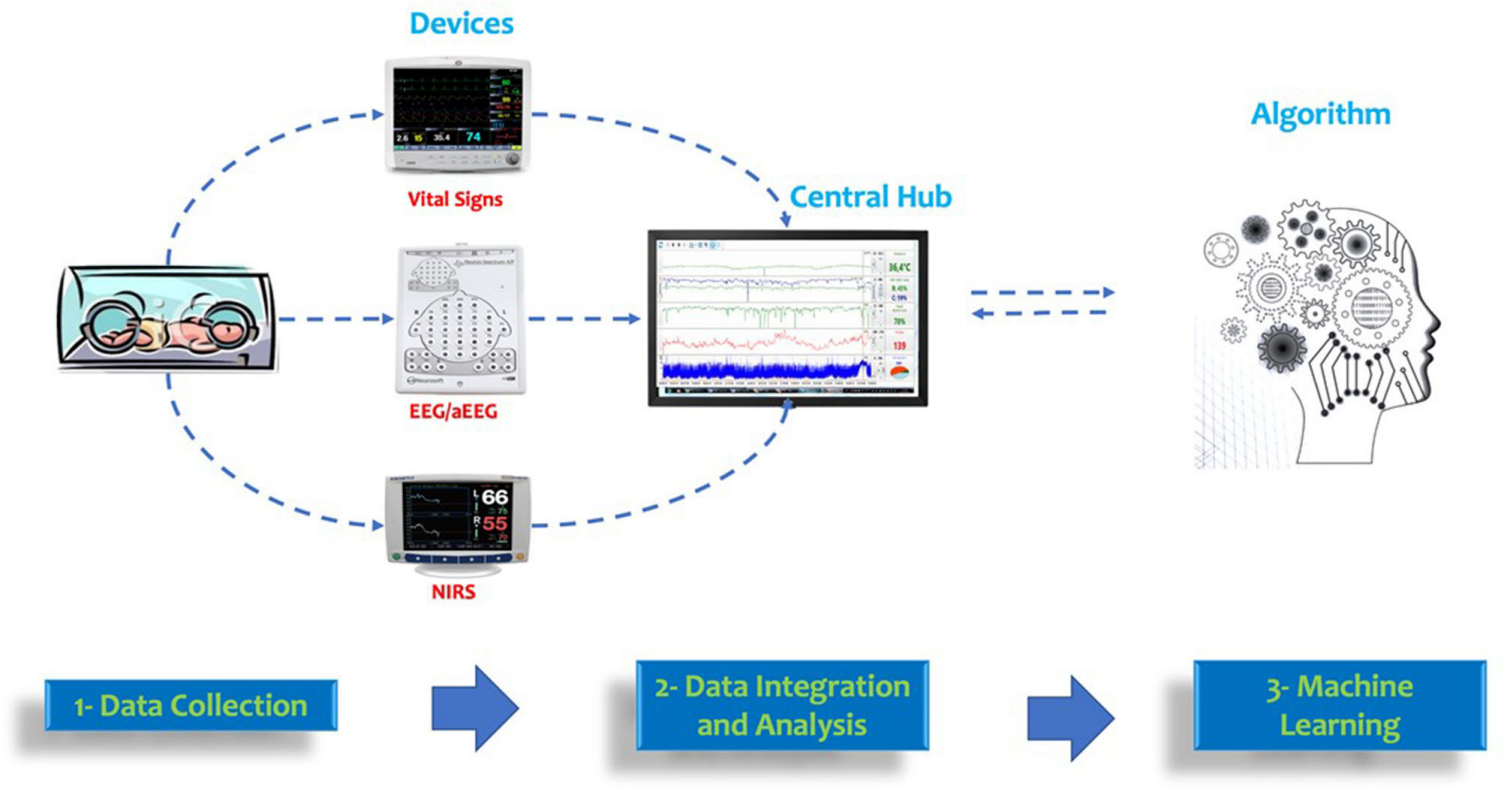
Overview of data collection and integration for multimodal monitoring in the NICU. Data from vital signs, EEG/aEEG, and NIRS devices can be integrated into a central hub. This data can be used for machine learning algorithms using continuous monitoring data.

Figure 3 represents an overview of data collection and integration for multimodal monitoring in the NICU.

## Artificial Intelligence and Machine Learning

With the advancement in computing power, data storage, and processing capabilities, artificial intelligence (AI) enables computer systems to perform activities that would typically require human intelligence and that people deem “*clever*.” AI is a term that refers to a system that is endowed with human-like intellectual processes, such as the capacity to reason, discover meaning, generalize, and learn from prior experience (87).

AI has been used for drug development, individualized diagnostics and treatments, molecular biology, bioinformatics, and medical imaging in healthcare. Additionally, AI systems can decipher illness patterns by scrutinizing and analyzing enormous volumes of data in electronic medical records.

Machine learning (ML) is a subfield of artificial intelligence in which computer algorithms learn to build predictive models from a given dataset. The main difference between ML and classical statistics is that ML aims to create the best possible predictive model from a training dataset, leveraging a test dataset to validate its results. Statistics aims to infer and validate relationships between variables on the whole dataset and are not optimized for its predictive capabilities. There are two broad categories of ML frameworks: supervised and unsupervised.

### Supervised Machine Learning

Supervised ML aims to create an algorithm capable of predicting an individual output given a specific input. In other words, the machine is shown instances of both input (x) and output (y), such that y = f(x). ML is dependent on large data sets containing several examples of how one or more input variables are related to a particular output. The expectation is that the resulting algorithm will make accurate predictions when confronted with new and previously unseen data. When big datasets are used to train and evaluate the system, supervised learning needs a significant amount of human labor (83).

Supervised learning can be used to predict both continuous and discrete values. For example, the expected heart rate of a patient in the next hour of monitoring is a continuous numerical value, and regression models should be used for prediction. In contrast, when building an automated seizure detection tool, the algorithm must learn from a binary input, either if a seizure is detected or not. For that kind of issue, classification models can be applied.

The choice of the model to apply to the desired task is a crucial step in developing predictive algorithms. For supervised learning, dozens of different models can be applied, including decision trees and deep learning, both widely used in the ICU for seizure detection, mortality prediction, phenotypic discovery, and disease prediction (88, 89).

### Unsupervised Machine Learning

No definitions are provided to the algorithm on how to process the data in this method of ML. Thus, the computer is expected to extract information from a vast set of unclassified data using either a set of rules or an unknown output. Given the absence of label information, a significant problem for the investigator when evaluating an unsupervised algorithm is determining the usefulness of the findings or determining whether the desired output was obtained. In addition, explainability may be challenging. However, unsupervised algorithms may be sufficiently efficient in exploratory attempts to comprehend vast sets of data. Clustering, anomaly detection, and dimensionality reduction are the most often utilized approaches (88).

**Figure 4:**
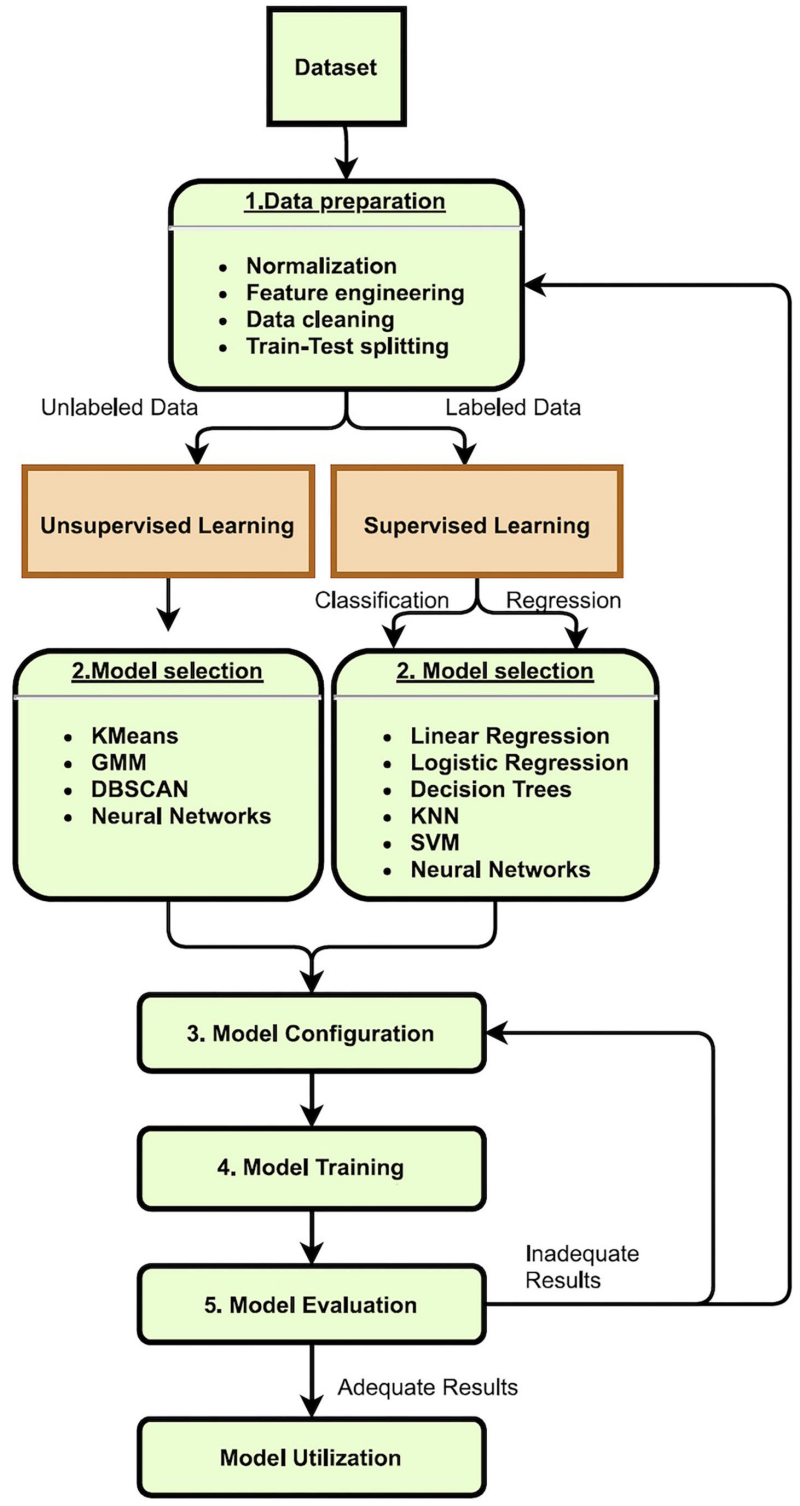
Machine learning pipeline: (1) Data preparation is the step where modifications to the dataset are made to improve the final results. That includes transforming, cleaning, and creating new features (feature engineering). In this step, the train-test data splitting can also be defined. (2) Model selection is the step where the appropriate model is chosen. That can significantly vary according to the dataset characteristics (like size and dimensionality) and the target variable. (3) Model configuration is the step in which all the parameters of the model must be set. (4) Model training is the step in which the machine finds the best-fitting predictive model according to the train dataset and model parameters. (5) Model evaluation is the step in which the user will analyze the results, mainly the train and test error of the generated predictive model.

Clustering algorithms are assigned to identify or partition huge data sets into subgroups and patterns with common characteristics. The algorithm is tasked with detecting unusual patterns in the dataset, such as outliers. When analyzing data with a large number of features, or dimensions, dimensionality reduction is advantageous. These algorithms may simplify the data by summarizing its main features and making it more understandable to humans and other ML algorithms. A machine learning pipeline is shown in Figure 4.

## AI Applications in Critical Care

There are multiple opportunities to implement AI in the medical context. Massive quantities of data contained in electronic medical records have been analyzed using unsupervised ML algorithms. Models have been created to extract critical information from medical files and identify individuals with a high cost of care (89). Supervised ML methods have demonstrated their value in radiography and histopathology due to their ability for automatic pattern detection of pictures (90). ML has been extensively utilized in the disciplines of surgery, particularly as it relates to robotics, cardiology, and cancer research to categorize tumor types and growth rates (91, 92).

Although ML is still in its inception in the ICU, multiple papers have already been published demonstrating its application in the care of critically ill patients. Several researchers have utilized massive population datasets to forecast duration of stay, ICU readmission, and death rates, as well as the likelihood of acquiring diseases such as sepsis or respiratory distress syndrome (93–95). Several other studies used smaller clinical and physiological datasets to help monitor patients receiving ventilatory support (96).

## AI in the Neonatal ICU

The NICU is a place where life-altering decisions are constantly made. Neonatologists collect data from various sources to create a picture of a newborn's status to guarantee they receive the best medical treatment available. Highly trained professionals utilize their judgment in conjunction with a continuous stream of patient data to ensure that as many infants have the best possible outcome. The use of AI could enhance the decision-making process and lead to better outcomes. Below we describe some applications of AI in the NICU.

### Diagnosis of HIE

Clinical diagnosis of asphyxia involves analysis of arterial blood gas and a standard neurological exam. Infants diagnosed with moderate to severe HIE meet the entry criteria for cooling, but new evidence show that neonates with mild HIE may also be at risk for disability (97). Precise identification of populations at high risk poses a clinical challenge in the field. A few approaches using AI have been studied to address this issue.

O'Boyle et al. published a study validating potential metabolites and evaluating their capacity to predict HIE independently and combined with clinical data. Term neonates with symptoms of perinatal asphyxia, with and without HIE, and matched controls were recruited prospectively at birth from two large centers. The study enrolled 511 infants, and by using logistic regression modeling and ML approaches, the optimal set of clinical and metabolite characteristics capable of predicting the development of HIE was found (98). Fifteen of twenty-seven potential metabolites revealed substantial changes in babies with perinatal asphyxia or HIE. Lactic acid and alanine were the significant metabolite predictors of HIE development, with an area under the curve (AUC) of 0.96 when combined with clinical data (95% CI, 0.92–0.95) (97).

EEG signal monitoring is known to be a valuable technique for identifying possible biomarkers of HIE (94). Over the last two decades, researchers throughout the world have developed a range of signal processing techniques for automated analysis and diagnosis of disease by examining aberrant activity using various forms of EEG recordings (99–101).

Temko et al. investigated an automated grading system (AGS) based on a multi-class linear classifier that graded short-term EEG epochs and converted them to a long-term EEG grade using a majority vote procedure (100). The AGS employed summary measurements of two sub-signals derived from a quadratic time-frequency distribution: amplitude modulation and instantaneous frequency. When compared to human assessment of the EEG, the four-grade AGS exhibited an accuracy of 83%. The features calculated from the generated sub-signals were shown to be more accurate in grading the EEG than measures based purely on the EEG, and the addition of new sub-grades based on EEG states to the AGS enhanced performance as well.

Utilizing the infant cry to diagnose asphyxia may present a novel approach for developing an accessible diagnostic tool. Previous studies have hypothesized that breathing difficulty resulting from asphyxia alters the patterns in the cry waves of affected infants, primarily attributed to the fact that speech and breathing are controlled by the same underlying physiologic process (102, 103).

*Ubenwa* is an application that uses voice recognition algorithms to identify early indications of birth asphyxia in a newborn's cry. A machine-based learning model capable of accurately classifying recordings of known asphyxiated children reported an accuracy of 89% in a laboratory environment. In 2017, a prototype mobile application was developed based on this concept with the potential to become a widely accessible solution even in low-resource settings (104).

### Automated Seizure Detection

Despite the availability of cEEG, reliable newborn seizure diagnosis remains challenging in clinical practice. Since 1992, techniques for automatically detecting newborn seizures in the EEG have been published (105). Significant advancements in data acquisition techniques, along with advances in computer technology, have substantially increased the accuracy of automated seizure detection approaches, and numerous research groups have developed and validated seizure detection algorithms for the newborn population (37, 106). The development of user-friendly ML-based software may facilitate the integration of automated EEG technology into the NICU, potentially increasing diagnostic accuracy and speed. A few of these algorithms have been included into commercially accessible EEG or aEEG systems (37, 106, 107).

The Efficacy of Intravenous Levetiracetam in Neonatal Seizures trial (NEOLEV2) used Persyst, a commercially available software, to evaluate participants' cEEGs (108). The software provided real-time seizure detection, which resulted in a higher detection rate, but failed to alleviate neurologists' burden, requiring human assessment due to low accuracy. RiskSLIM is a sparse linear integer machine with a high prediction accuracy (AUC = 0.83; similar to other widely used ML algorithms) (108).

Lawrence et al. studied a cohort of 40 encephalopathic infants for 72 h using a limited-channel aEEG and a software-based seizure event detector. Offline evaluation of EEG data was conducted independently. During an average of 68 h of monitoring, twenty-five babies developed EEG seizures on a limited channel aEEG, and 1,116 EEG seizures were detected retrospectively. 615 (55%) of the seizures were recognized in real-time by the seizure detection algorithm. Seizure detection software accuracy increased with seizure duration, up to 73% for seizures lasting more than 30 s and 87% for seizures lasting more than 60 s. Seizure detection had a false-positive rate of 1 event every 11 h of monitoring, resulting in a positive predictive value (PPV) of 73% and a negative predictive value (NPV) of 99% (37).

In a large randomized controlled trial conducted in eight centers in Europe, the diagnostic accuracy of an automated seizure detection system named Algorithm for Neonatal Seizure Recognition (ANSeR) was assessed. This study included 264 babies between the age of 36 and 44 weeks with or at high risk of seizures requiring EEG monitoring. One arm of the trial used cEEG plus ANSeR, connected to the EEG monitor and displayed a real-time seizure likelihood trend. In contrast, the other arm received only cEEG monitoring. Electrographic seizures occurred in 25% of newborns in the algorithm group compared to 29.2% in the non-algorithm group. Despite not enhancing the identification of individual neonates with seizures, the algorithm group correctly identified a greater proportion of seizure hours when compared to the non-algorithm group (109, 110).

There is a growing number of papers evaluating automated seizure detection algorithms in the NICU. Studies from the last five years are displayed in Table 2.

**Table 2:** List of studies using ML for automated seizure detection published in the last 5 years.

**References**	**Objective**	**Study population**	**Analyzed parameters**	**Outcomes**	**Conclusion**
Ansari et al. (111)	To describe a multi-stage classifier method for enhancing an automated EEG-based neonatal seizure detector.	71 Term Neonates with HIE or suspicion of seizures	EEG-polygraphy data	- The proposed post-processor (when sensitivity threshold = 0.3) decreased FAR by 64%, whereas the GDR was reduced by 7%.	A significant improvement of a previously developed automated neonatal seizure detector was achieved by combining a machine learning technique with the heuristic algorithm.
				- Identifying seizures lasting less than 30 s remains the most challenging task of the post-processor since it includes 26% of true and 60% of false detections.	
Ansari et al. (112)	To improve the overall performance of a previously developed multi-stage neonatal seizure detector, particularly by improving the performance of the short seizure detections.	48 Neonates with HIE	EEG-polygraphy data	- Almost all seizures longer than 1min were detected by both methods, while the short seizures are still not entirely detected.	An adaptive learning method was proposed to decrease the false alarm rate and increase the positive predictive value of a previously developed multi-stage neonatal seizure detector, particularly for very short seizures and false alarms.
				- The number of false detections of very short seizures (<30 s) decreased by 50%	
				- The PPV of very short seizures also increased from 41 to 59% by the proposed method (higher reliability of the alarms).	
Ansari et al. (113)	To use deep CNNs and random forest to optimize feature selection and classification automatically.	48 Neonates	EEG recordings	- AUC of the CNN and RF method is 8% higher than the pure CNN with the fully connected network (83 vs. 75%).	The main advantage of the proposed method is that it does not require a hand-engineered feature extraction process. Still, it automatically extracts the required features and optimizes them based on the training data.
				- CNN's specificity was 5% lower, while the averaged false alarm rate per hour is 0.04 better than those of the heuristic methods.	
Bogaarts et al. (114)	To gain insight into optimal training set composition for age-independent seizure detection and compare classification performance, specific properties of the classifiers themselves.	39 Neonates with post-conception age ranging from 28 to 59 weeks / 39 adults	EEG recordings	- With FBC, the amount of neonatal SVs increased to 55%.	Adult and newborn patients can both benefit from an age-independent SVM seizure detection system. However, it is critical that EEG data from each age group be utilized for training the classifier.
				- For newborn seizures detection, the classifier trained only on adult EEG data performed considerably worse than the classifier trained on neonatal EEG data or the one trained on both neonatal and adult EEG data.	
Mathieson et al. (115)	To describe a novel neurophysiology-based performance analysis of automated seizure detection algorithms for neonatal EEG to characterize the features of detected and undetected seizures and the causes of false detections to identify areas for algorithmic improvement.	20 Term neonates	EEG recordings and ANSeR SDA	−421 seizures were initially detected in a total of 1,262.9 h of EEG (mean 63.1).	The analysis presented has elucidated several aspects of the performance of the SDA from a neurophysiological perspective. The analysis of the ANSeR algorithm highlighted many areas for possible improvement, which have since resulted in increased performance in the ANSeR algorithm's beta version.
				- Clinical neurophysiologists confirmed seizures in 419 of the 421 events annotated by experienced electroencephalographers (99.76%).	
				- More seizures were detected at lower thresholds (higher sensitivity), but the false detection rate is also higher.	
				- False detection rates between seizure and non-seizure neonates were not statistically different at any of the three thresholds tested (threshold 0.4, *p* = 0.579, threshold 0.5, *p* = 0.280, and threshold 0.6, *p* = 0.218).	
				- For all three thresholds tested, 8/10 of the seizure features were a significant predictor of automated seizure detection.	
				- The AUCs (95% CI) for the multivariate model at all 3 ANSeR sensitivity thresholds was significantly better (threshold 0.4 *p* < 0.001, threshold 0.5 *p* < 0.001, threshold 0.6 *p* = 0.023) than the highest AUC in the corresponding univariate analysis (seizure duration).	
Mathieson et al. (116)	To validate the performance of the neonatal SDA on a more extensive database of unseen, unedited, continuous, multi-channel EEG data from 70 term newborns collected at two sites	70 Term neonates	EEG recordings and SDA	- There is variability in seizure detection and false detection rates across babies.	The potential of the SDA to support clinical decisions regarding AED administration was shown in this study. The study has validated a neonatal SDA on a large EEG dataset and demonstrated that it achieves a clinically useful level of seizure detection with acceptable false detection rates.
				- The highest performing threshold varies depending on the parameter of interest.	
				- There is a trade-off between the number of seizure and non-seizure babies detected depending on the SDA sensitivity threshold.	
				- The best performing SDA sensitivity threshold was at 0.8 (30/35 seizure babies identified, 31/35 non-seizure babies identified).	
				- The maximal level of agreement was at a sensitivity threshold of 0.4.	
				- The median AUC for the validation study, estimated on neonates with seizures, was 0.945. The mean AUC was 0.933.	
Mathieson et al. (117)	To evaluate the morphology of seizures in newborns before and after phenobarbital treatment and assess the influence of any variations on automated seizure detection rates.	18 Term neonates	EEG recordings	- No significant differences between groups were found in seizure duration, rhythmicity, frequency variability (over the whole seizure), background EEG grade, seizure waveform morphology at the start or peak of the seizure, or seizure waveform morphology change from start to a peak of a seizure.	Phenobarbital reduces the amplitude and propagation of seizures, but ANSeR performance is unaffected by these changes.
				- The seizure detection rates (sensitivity threshold 0.3) were not significantly different, with a median detection rate of 77% for pre-phenobarbital seizures and a 73% detection rate for post-phenobarbital seizures.	
Temko et al. (118)	To propose a probabilistic framework for semi-supervised adaptation of a generic patient-independent neonatal seizure detector.	18 Full-term neonates	Continuous neonatal EEG recordings	The Oracle patient-dependent system's performance, patient-dependent PD-GMM is 97.51 and 86.33% for AUC and AUC90, respectively.	A combination of patient adaptive generative and patient independent discriminative classifiers has improved the detection of neonatal seizures throughout long EEG recordings. More accurate detection comes from the different nature of the classification approaches and the real-time incorporation of patient-specific data.
				- The patient adaptive GMM system (PA-GMM) provides a performance that improves over its patient-independent GMM counterpart (PI-GMM) _ 96.91 vs. 95.70% for AUC, and 82.6 vs. 78.4% for AUC90.	
Tapani et al. (119)	To estimate several features based on the SNLEO and use machine learning to optimize the SC method.	79 Term neonates with multiple etiologies.	Continuous multi-channel EEG recordings	- SNLEO features alone resulted in a median AUC of 0.963 (IQR 0.919–0.985), significantly higher than the original SVM-based method (*p* = 0.024).	By using SNLEO features adapted from the SC technique, the performance of an SVM-based neonatal EEG seizure detector is significantly improved.
				- The SNLEO method was significantly improved by incorporating a selected number of features from the SVM-based detector (*p* = 0.002). Median AUC using this feature set was 0.981 (IQR 0.942–0.994).	
Tapani et al. (120)	To develop methods for detecting the non-stationary periodic characteristics of EEG seizures by adapting estimates of the correlation both in the time SC and time-frequency domain TFC.	79 Term neonates.	EEG recordings	- The proposed measures were very discriminative in detecting seizures (median AUCSC: 0.933).	The suggested SDA surpasses their implementation of leading techniques across all concatenated EEG recordings. There is still room for the development of features for neonatal SDAs, emphasizing time-varying methods.
				- When applied to multi-channel recordings, the resulting SDA achieved a median AUC of 0.988 compared to consensus annotations, outperformed two state-of-the-art SDAs (*p* = 0.001), and was non-inferior to the human expert in 73/79 of newborns.	
Stevenson et al. (121)	To combine two recently developed NSDAs, including the hybrid algorithm combining the feature with the output of the CNN using a kernel SVM, for improvement of detection performance.	79 Neonates	EEG recordings	- Increasing the minimum seizure duration from 10 to 30 s provides the most significant increase in performance with the highest SDR and lowest FD/h.	Automated approaches for detecting newborn EEG seizures are accurate, possibly offering physicians in the NICU with reliable interpretations.
				- The area under the receiver operator characteristic of the NSDA was 0.952 compared to the expert consensus annotation (95% CI: 0.0927–0.971).	
				- The inter-observer agreement (IOA) of seizure identification was not significantly different between the NSDA and human analysis and was further improved by increasing the minimum seizure length from 10 s to 30 s.	
Pavel et al. (110)	To study the ANSeR algorithm's real-time performance in a multi-center study by comparing diagnostic accuracy to identify electrographic seizures with and without the use of ANSeR as a bedside support tool for clinicians.	258 Neonates between the correct gestational age of 36 and 44 weeks	EEG recordings and ANSeR	- The primary outcome measure of diagnostic accuracy (sensitivity, specificity, and false detection rate) was not statistically different between the two groups for detecting an infant with seizures.	Although all participating hospitals were experienced in neonatal EEG and the clinical teams were generally comfortable interpreting the aEEG or cEEG, the support provided by the ANSeR algorithm still had a considerable effect on the seizure recognition rate. The study suggests that the benefit provided by the ANSeR algorithm might be more significant if it was made available to centers with less experience of interpreting neonatal EEG at the cot side.
				- The percentage of seizure hours identified was higher in the algorithm group (177 [66.0%; 95% CI 53.8–77.3] vs. 177 [45.3%; 34.5–58.3]; difference 20·8% [3.6–37.1]).	
				- The false detection rate on the seizure record form did not differ between the groups.	
				- No significant differences were found between the groups regarding the secondary outcomes of seizure characteristics (total seizure burden, maximum hourly seizure burden, and median seizure duration) and the percentage of neonates with seizures given at least one inappropriate antiseizure medication.	

*FAR, false alarm rate; GDR, good detection rate; PPV, positive predictive value; CNN, convolutional neural networks; RF, random forest; FBC, feature baseline correction; SVs, support vector; SVM, support vector machine; ANSeR, algorithm for neonatal seizure recognition; SDA, seizure detection algorithm; AUC, area under the curve; AED, antiepileptic drug; GMM, Gaussian mixture model; SNLEO, smoothed non-linear energy operator; SC, spike correlation; TFC, time-frequency correlation; NSDAs, neonatal EEG seizure detection algorithms; SDR, seizure detection rate; FD/h, false detections per hour*.

### Prediction of Mortality

Numerous ML models have been reported in adult research, including those that predict death in a variety of disease processes (87, 88). However, there are much fewer AI models reported in pediatrics and neonatology to date. A recent systematic review included eleven studies examining the prediction accuracy of AI models for newborn mortality. This research included a total of 1.26 million babies born between 22 weeks and term age. The mean AUC ranged from 58.3 to 97.0 percent in this review, indicating their capacity to predict neonatal death. The most common features used in the predicting models are birth weight, gestational age and APGAR scores. Other standard features include pH, blood pressure, use of prenatal steroids, and multiple births (113).

### Early Recognition of Sepsis

Sepsis is a leading cause of newborn mortality. The commercially available “HeRO” monitor analyzes ECG data from current bedside monitors for reduced heart rate variability and brief decelerations associated with sepsis and transforms them to a score (the HRC index or HeRO score). This score represents the chance of a patient having clinical deterioration due to sepsis within 24 h. Displaying the HeRO score reduced mortality by more than 20% in a randomized study of 3003 very low birth-weight babies. Current research attempts to integrate respiratory and heart rate data to enhance ICU care (122).

### Predicting Extubation Failure

Given the independent association between mechanical ventilation and significant unfavorable outcomes, efforts should be taken to restrict its duration. However, existing methods for identifying extubation readiness are ineffective, and a large proportion of newborns fail extubation and require reintubation, a procedure that may result in increased morbidity. Numerous objective metrics have been proposed to improve the definition of the ideal time for extubation, but none have been demonstrated to be clinically helpful.

A recent multi-center diagnostic study investigated data from 259 newborns at five neonatal intensive care units over 5 years as part of the prospective Automated Prediction of Extubation Readiness (APEX) project (123). The study included 274 intubated neonates less than 1,250 g who were considered ready for extubation and had ET-CPAP before extubation. Cardiorespiratory signals were recorded electronically for 5 mins before extubation, and signs of clinical instability were monitored (124).

The data imply that extremely preterm newborns frequently exhibit clinical instability during ET-CPAP and that clinical event combinations were insufficient to define Spontaneous Breathing Trials (SBT's) accurately. As a result, the authors concluded SBTs might offer minimal value to the evaluation of extubation readiness.

### Predicting Gestational Age

Particularly in low-middle income countries, the last menstrual period is frequently considered an inaccurate estimate of gestational age at birth (125). Using AI for prediction might be an interesting approach to minimize this effect. Models to evaluate pregnancy status were built to predict gestational age when the last menstrual period and fetal anatomical evaluation were not accessible (126).

A recent study involving 1318 newborns from Africa and Asia found that applying ML to birth weight and new-born metabolomic screening data can improve postnatal prediction of gestational age (GA) at birth. 85.21 % (95% CI 72.31–94.65) of GAs were properly predicted to within one week. The model also showed high sensitivity (100%) and specificity (92.6%) for differentiation of preterm and term birth (127). Meenakshi et al. (128) showed good results of a model to automatically estimate the GA at third trimester by using biparietal diameter and kidney length.

Additional ML approaches were also studied in the NICU, including automatic detection of sleep-wake-cycling for prediction of neurodevelopmental outcomes (129–132).

## Telemedicine

Telemedicine is defined by the World Health Organization (WHO) as “the delivery of health care services by all health care professionals using information and communication technologies for the exchange of valid information for the diagnosis, treatment, and prevention of disease and injury, research and evaluation, and continuing education of health care professionals” (133). Additionally, four critical components of telemedicine were identified: (i) provision of clinical support; (ii) purpose to overcome geographic barriers; (iii) the use of a variety of information and communication technology; and (iv) the objective of improving population health (134). This model represents the natural growth of healthcare in the digital era, reducing distances between distant places, increasing access and reach of specific techniques, and decreasing structural costs. In education, it can be used to facilitate learning through live interactive audiovisual connections, live video broadcast, or viewing online-stored educational materials (133–136).

Telemedicine has the potential to play an essential role in providing specialized care to remote populations with limited resources. Centralized systems may communicate in real-time with a large number of centers, leveraging educational activities, consulting, and monitoring to improve service quality. Protecting Brains and Saving Futures (PBSF) is a Brazilian program that has already connected 35 hospitals in the country. Neonates undergoing EEG or aEEG and NIRS monitoring are assisted by a remote team in a monitoring center, using encrypted data to ensure the privacy of sent information. This approach may also be used in research contexts, where studies done in low-middle income countries (LMIC) might benefit from teams' assistance in developed countries, potentially improving the quality of data obtained substantially. The Prevention of Epilepsy by Reducing Neonatal Encephalopathy (PREVENT) study (NCT04054453) used a similar method to monitor and collect data from newborns in three sites in India. All EEG data is securely sent to a cloud-based server. Cost-effectiveness analysis and legal and regulatory concerns are significant challenges to this approach in LMICs.

**Figure 5:**
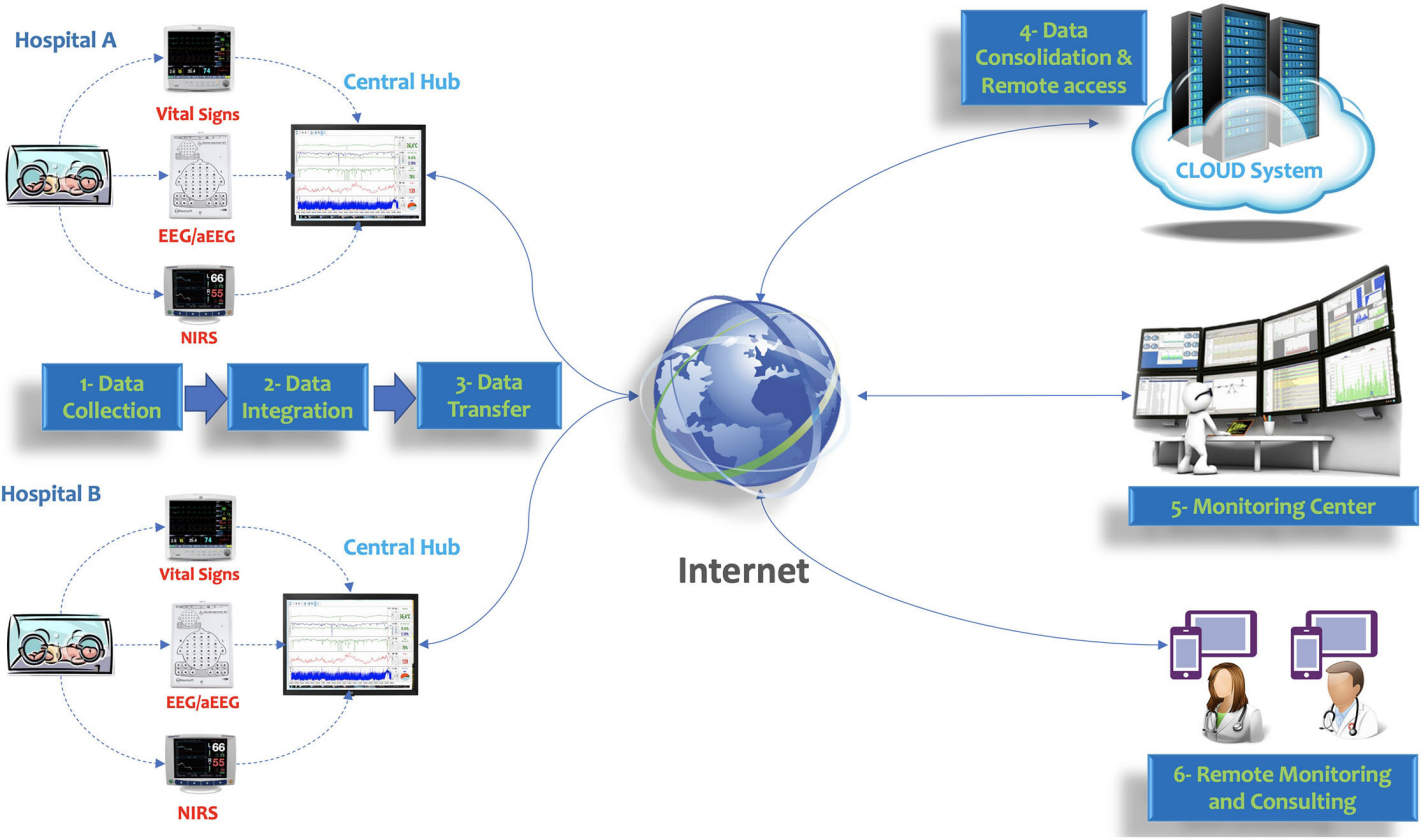
Model of architecture for multimodal monitoring accessed by telemedicine approach. Vital signs, EEG/aEEG, and NIRS are integrated using a central hub. The encrypted data is transferred to the cloud system and accessed by a monitoring center or remote assistance.

A model of architecture for multimodal monitoring accessed by telemedicine approach is shown in Figure 5.

## Futures Directions and Conclusions

The creation of the Neuro-NICU demonstrates a genuine potential for closing the gap between survival and improved neurodevelopmental outcome. Given the increased emphasis on neurocritical care, both aEEG/EEG and NIRS have demonstrated value as bedside monitors in the NICU. Multimodal brain and hemodynamic monitoring in newborns are possible, safe, and well-tolerated. Further research should be conducted to determine the impact of this strategy in a more extensive range of clinical scenarios, emphasizing evidence to improve clinical outcomes.

Tele-health is increasingly used to assist remote centers with education, monitoring, and consulting (137). The combination of multimodal monitoring and telemedicine may improve understanding of physiology and provide a vast repertoire of data.

With the advancement of processing power and the growth of data availability, ML algorithms are proving to be an interesting tool in ICU research. However, one of the primary challenges to adopting ML in clinical practice is the ethical considerations of depending on ML for clinical decision-making. While ML has been proven to surpass traditional methods for clinical decision-making, there is significant concern over who is responsible if an ML makes a mistake.

One of the key challenges in clinical research is gathering and evaluating big datasets for prospective studies. Only a small percentage of previously published studies verified their model using an independent cohort.

In conclusion, neonatology is one of the medical specialties that have scientifically advanced the most in recent decades and today is focused on preserving the quality of life in high-risk infants. The broader use of brain monitoring, associated with intelligent analysis of large amounts of information collected on-site or remotely, has the potential to significantly change clinical management and neonatal outcomes within the next 10 years.

## Author Contributions

GV conceived the idea and wrote the manuscript. DR, MM, and MJM reviewed, edited, and provided contributions to the manuscript. JC provided critical feedback and support on data integration and artificial intelligence topics. All authors contributed to the article and approved the submitted version.

## Conflict of Interest

The authors declare that the research was conducted in the absence of any commercial or financial relationships that could be construed as a potential conflict of interest.

## Publisher's Note

All claims expressed in this article are solely those of the authors and do not necessarily represent those of their affiliated organizations, or those of the publisher, the editors and the reviewers. Any product that may be evaluated in this article, or claim that may be made by its manufacturer, is not guaranteed or endorsed by the publisher.
